# Beyond Methotrexate and Biologics in RA – Efficacy of JAK Inhibitors and their Place in the Current Treatment Armamentarium

**DOI:** 10.31138/mjr.31.1.120

**Published:** 2020-06-11

**Authors:** Katerina Chatzidionysiou

**Affiliations:** Department of Medicine, Solna, Karolinska Institutet, Rheumatology Unit, Karolinska University Hospital, Stockholm, Sweden

**Keywords:** JAK inhibitors, methotrexate, biologics, treatment, rheumatoid arthritis

## INTRODUCTION

Treatment of rheumatoid arthritis (RA) has transformed dramatically during the last decades. A substantially revised treatment paradigm, comprising treat-to-target strategies, earlier initiation of treatment and the development and introduction to clinical practice of immune-targeted therapeutics based upon pathogenesis driven principles, has contributed to this dramatic progress.^1^ New and highly effective disease modifying anti-rheumatic drugs (DMARDs) are introduced as soon as possible after diagnosis is confirmed in order to inhibit radiographic progression due to irreversible joint damage that causes functional disability, chronic pain, early unemployment, and poor quality of life. Today, many conventional synthetic DMARDs (csDMARDs) and biologic DMARDs (bDMARDs) are available with distinct mechanisms of action.

Despite this dramatic progress, however, rates of remission remain low. In a real-life RA study, remission rates did not exceed 20%.^2^ Many patients do not respond at all (primary inefficacy) or lose the effectiveness to one or more bDMARDs, which can be at least partly explained by pharmacokinetic factors in which anti-drug antibodies and the drug form immune complexes that lead to abrogation of pharmacological activity of the drug and/or enhanced drug clearance. A number of studies from clinical practice indicate that as many as 50% of all patients discontinue their TNFi treatment during the first 3 years.^3,4^ Observational studies have also demonstrated a reduced effectiveness of various bDMARDs parallel to the increasing line of therapy.^5,6^ Additionally, a troubling minority are truly difficult to treat, failing multiple different mechanisms of action, posing a significant unmet need in the RA treatment field.^7^

## MECHANISM OF ACTION OF JAK INHIBITORS

During the last years, a third category of DMARDs has appeared, the targeted synthetic DMARDs (tsDMARDs), consisting of the Janus Kinase (JAK) inhibitors. The JAK family comprises four members: JAK1, JAK2, JAK3 and TYK2. They are cytoplasmic tyrosine kinases that mediate the intracellular signalling by association with type 1 and type II cytokine receptors.^8^ JAK activation leads to activation of their downstream substrates, the signal transducer and activator of transcription (STAT) proteins, followed by their nuclear translocation and subsequent activation of target genes.^9^ The JAK/STAT pathway plays a crucial role in cellular signalling pathways for a wide array of cytokines and growth factors, thus regulating the immune and inflammatory process.^10^ JAK activation stimulates cell proliferation, differentiation, cell migration and apoptosis. These cellular events are critical to haematopoiesis, immune development, adipogenesis, sexually dimorphic growth, and other processes. Mutations that reduce JAK/STAT pathway activity affect these processes.^11^

**Figure 1.**
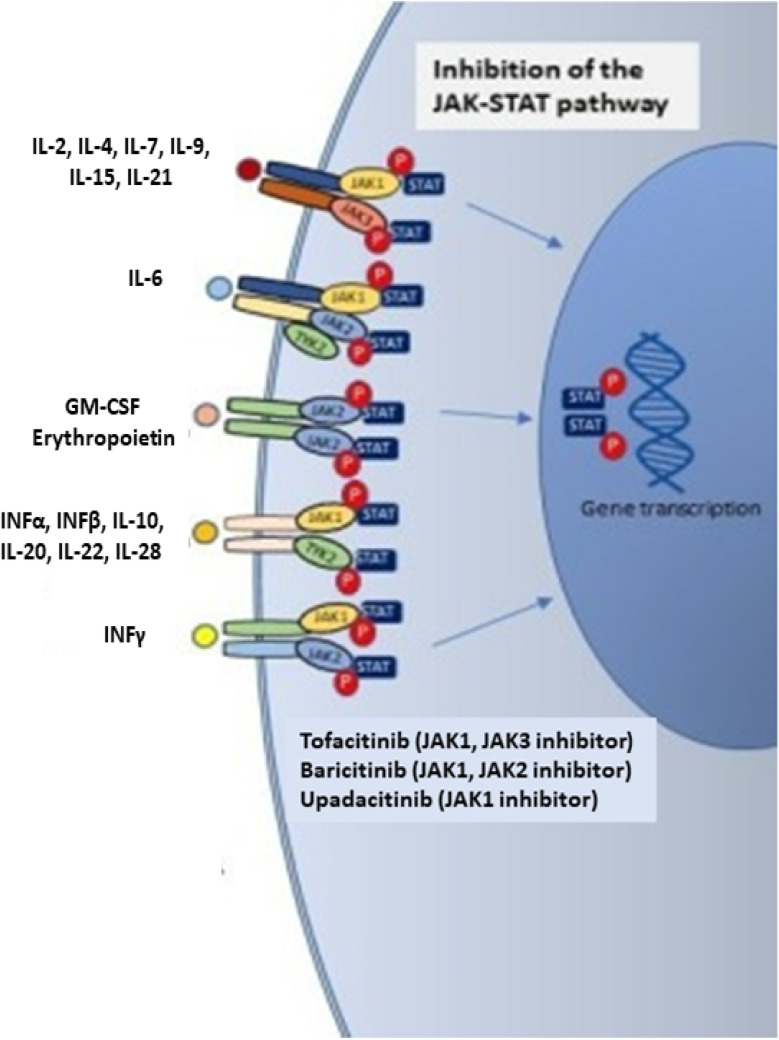
Biologic DMARDs and targeted synthetic DMARDs approved for the treatment of rheumatoid arthritis. DMARDs, disease-modifying antirheumatic drugs; IFN, interferon; IL, interleukin; IL6R, interleukin 6 receptor; INFγ, interferon γ; JAK-STAT, Janus Kinase/Signal Transducer and Activator of Transcription proteins; mAb, monoclonalantibody; MHC, major histocompatibility complex; mIL6R, membraneinterleukin 6 receptor; sIL-6R, soluble interleukin 6 receptor; TCR, T cellreceptor; TNF, tumor necrosis factor.

Since many type I and II cytokines that exert their function through the JAK-STAT pathway are involved in RA pathogenesis, JAK targeting may cause immunosuppression offering a new approach to RA treatment. In contrast to bDMARDs, which are large proteins that can only be administered parenterally, JAK inhibitors are small molecules that are orally available and can cross the cell membrane to block activity of one or more cytoplasmic JAKs (*Figure 1*). They are non-immunogenic and have a shorter half-life than biologics, with the advantage of more rapid reversal of any drug-related adverse events. In *Figure 1*, the biologic and targeted synthetic DMARDs approved for RA are summarized.

## EFFICACY OF JAK INHIBITORS

Three JAK inhibitors have hitherto been approved by the United Stated Food and Drug Administration (FDA) and the European Medicines Agency (EMA) for the treatment of RA. Tofacitinib, which was the first JAK inhibitor to be approved, selectively inhibits JAK1 and JAK3, while baricitinib is a selective JAK1/2 inhibitor. Just a few months ago, the third drug of this category was approved by both the FDA and the EMA, upadacitinib. Upadacitinib is the first selective JAK1 inhibitor approved. All the above JAK inhibitors have undergone extensive clinical trials and have demonstrated significant efficacy and acceptable safety profile. Here, we will try to summarize the phase III clinical trials for the three drugs in different patient populations, depending on the previous DMARD treatment.

### 1. csDMARDs naïve RA population

In ORAL Start, a 24-month, phase III, randomized controlled trial, 958 MTX-naïve RA patients with active RA were randomized to tofacitinib 5 mg bd, 10 mg bd, or methotrexate (MTX) (target dose 20 mg/week).^12^ Tofacitinib monotherapy resulted in clinically and statistically significant American College of Rheumatology (ACR)70 responses at month 6, achieved by 25.5% in the 5 mg bd group and 37.7% in the 10 mg bd group, as compared with 12% of patients in the MTX group (*P* < 0.001 for both comparisons). Other primary outcomes included statistically significant improvements in physical function and inhibition of progression of structural damage compared with MTX. There were also statistically significant differences between both tofacitinib doses and MTX with respect to multiple patient-reported outcomes (PROs), such as Patient Global assessment (PtGA), pain, Health Assessment Questionnaire (HAQ), fatigue, Health Related Quality of Life (HRQOL).^13^

Baricitinib was evaluated as monotherapy or in combination with MTX and compared to MTX in DMARD naïve patients with active RA in RA-BEGIN, a 52-week, phase III, non-inferiority trial.^14^ 588 patients with active RA and no prior treatment with csDMARDs (no or limited exposure to MTX) or bDMARDs were enrolled in the study. The median disease duration of RA in this study was 0.2 years, significantly lower than the ORAL-start. Patients were randomized to baricitinib 4 mg monotherapy, MTX monotherapy, or combination baricitinib 4 mg and MTX. The primary end point was ACR20 at 24 weeks between baricitinib monotherapy and MTX monotherapy. At 24 weeks, ACR20 response was significantly higher with baricitinib monotherapy (77%) and combination (78%) compared with MTX monotherapy (62%). Baricitinib monotherapy even met the superiority criteria over MTX monotherapy. The rate of remission was 40% for baricitinib and 24% for MTX. Although radiographic progression was reduced in both baricitinib groups compared to MTX monotherapy, the difference was statistically significant only for baricitinib plus MTX and not for baricitinib monotherapy. Baricitinib alone or in combination with MTX, when used as initial therapy, resulted in significant improvement compared to MTX in the majority of the pre-specified PRO measures.^15^

Upadacitinib was evaluated as first line therapy in the SELECT-EARLY trial, a 48-week, double blind active comparator-controlled trial. In SELECT-EARLY, MTX-naïve patients with active RA who were positive for both RF and ACPA and/or had ≥1 joint erosion were randomized to once-daily upadacitinib at 15mg or 30mg, or weekly MTX. Separate primary endpoints were ACR50 at Wk12 (for the FDA approval), or the proportion of pts achieving DAS28CRP<2.6 at Wk24 (for the EMA approval). Secondary endpoints included mean changes from baseline in modified Total Sharp Score (mTSS) and proportion of pts with no radiographic progression (mTSS≤0) at Wk24. Around 950 patients were randomized, and approximately half of them had an RA diagnosis of <6 months and RA symptoms <2 years. Of the 945 pts, 874 (92.5%) had no prior MTX exposure; 706 (74.7%) had no prior csDMARD exposure. Both primary endpoints were met. Significantly more patients receiving upadacitinib vs MTX achieved ACR50 responses at week 12 (52.1% and 56.4% vs 28.3%) and DAS28CRP<2.6 at week 24 (48.3% and 50.0% vs 18.5%). At week 24, mean difference in mTSS was 0.14 and 0.07 vs 0.67; significantly more pts had no radiographic progression on UPA 15 and 30mg vs MTX. LDA and remission by various criteria at Wks12 and 24 were achieved in more pts on UPA vs MTX (nominal p<.001 for all).

**Figure 2.**
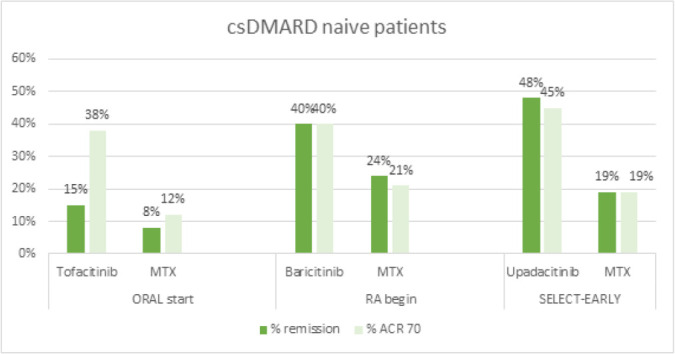
ACR70 response and remission rates (based on DAS28<2.6) for tofacitinib, baricitinib and upadacitinib in DMARD naïve RA patients. All three JAK inhibitors yielded significantly higher clinical efficacy compared to methotrexate (MTX).

In *Figure 2*, the most stringent clinical outcomes, namely ACR70 responses and remission rates (defined as DAS28<2.6) for the three JAK inhibitors are summarized. Differences observed across the three JAK inhibitors in efficacy in the same patient population (DMARD naïve) are difficult to interpret. Differences in study design and especially in patient and disease characteristics, such as disease duration, might explain at least partly the differences. Thus, we cannot make any assumptions or attempts to indirectly compare the three different JAK inhibitors.

### 2. After failure of MTX, bDMARD naïve population

Two RCTs evaluated the efficacy of tofacitinib in RA patients who have discontinued MTX and/or other cs-DMARDs due to inefficacy or intolerance. There are significant differences in study design in these trials. In the ORAL Standard, a 12-month, phase III trial, 717 patients who were receiving stable doses of methotrexate and were inadequate responders were randomly assigned to tofacitinib (2 different doses), the anti-TNF bDMARD adalimumab, or placebo.^16^ The three primary outcome measures were an improvement in ACR20 responses at month 6; the change from baseline to month 3 in HAQ-DI; and the percentage of patients meeting DAS28-4(ESR) remission criteria (<2.6) at month 6. At month 6, ACR 20 response rates were significantly higher among patients receiving 5 mg or 10 mg of tofacitinib (51.5% and 52.6%, respectively) and among those receiving adalimumab (47.2%) than among those receiving placebo (28.3%) (P<0.001 for all comparisons). There were also greater reductions in the HAQ score at month 3 and higher percentages of patients with a DAS28-4(ESR) below 2.6 at month 6 in the active-treatment groups than in the placebo group. It is important to underline that this trial was not designed to provide head-to-head comparative efficacy and should not be interpreted as evidence of tofacitinib superiority or non-inferiority to adalimumab.

There were clinically meaningful improvements for various important PROs with tofacitinib 5 and 10 mg bd and adalimumab that were significantly superior to placebo at 3 months and sustained to month 12.^17^

The ORAL Scan RCT focused on radiographic progression. It was a 24-month trial designed to determine whether tofacitinib has an effect on structural damage in RA patients with an inadequate response to MTX.^18^ 797 patients were randomized to tofacitinib 5 mg bd, 10 mg bd, or placebo. All patients had background MTX. At month 6, ACR20 response rates for tofacitinib 5 mg and 10 mg bd were higher than those for placebo (51.5% and 61.8%, respectively, versus 25.3%; both P < 0.0001). At month 6, least squares mean (LSM) changes in total modified Sharp/van der Heijde score for tofacitinib at 5 mg and 10 mg twice daily were 0.12 and 0.06, respectively, versus 0.47 for placebo (P = 0.0792 and P ≤ 0.05, respectively). Both doses of tofacitinib showed negligible change in mTSS. Statistical significance was achieved compared to placebo only for the higher dose of tofacitinib (10mg bd) but not with 5mg.

Regarding baricitinib, two large RCTs evaluated its efficacy and safety in a csDMARDs inadequate responders population of RA patients. In RA-BEAM, baricitinib was compared to placebo and adalimumab.^19^ It was a double-blind, randomized, double-dummy, placebo- and active-controlled, parallel-arm trial. 1307 RA patients with inadequate response to MTX were randomized to baricitinib, adalimumab or placebo. A prespecified multiple-testing procedure was used to control for type I errors related to the primary and major secondary objectives, including two assessments against adalimumab: a test of superiority with respect to DAS28-CRP and a test of noninferiority with respect to ACR20. A prespecified noninferiority margin of 12% was chosen. In the plan for multiple comparisons, if non-inferiority was shown, the superiority of baricitinib to adalimumab was evaluated. At week 12, more patients had an ACR20 response with baricitinib than with placebo (primary end point, 70% vs. 40%, P<0.001). Baricitinib plus MTX was also found to be non-inferior to adalimumab plus MTX for the ACR20 response, with a margin of 12% (70% *vs* 61% for adalimumab), and was therefore considered to be significantly superior to adalimumab (*P* = 0.01). All major secondary objectives were met, including inhibition of radiographic progression of joint damage, according to the mTSS at week 24 with baricitinib versus placebo (mean change from baseline, 0.41 vs. 0.90; P<0.001) and an increased ACR20 response rate at week 12 with baricitinib versus adalimumab (70% vs. 61%, P=0.014). There was no significant difference between baricitinib plus MTX and adalimumab plus MTX in inhibition of radiographic progression. Baricitinib also provided greater improvement in most PROs with statistical significance at several time points compared with placebo and adalimumab, including physical function, morning joint stiffness, pain, fatigue, overall work impairment and quality of life.^20^

In RA-BUILD, a phase III, double-blind 24-week study, 684 bDMARD-naïve patients with RA and inadequate response or intolerance to ≥1 csDMARDs were randomly assigned 1:1:1 to placebo or baricitinib (2 or 4 mg) once daily, stratified by region and the presence of joint erosions.^21^ In this study, around 25% of patients had failed 3 or more csDMARDs. More patients achieved ACR20 response at week 12 with baricitinib 4 mg than with placebo (62% vs 39%, p≤0.001), as well as several other clinical outcomes and PROs. In a supportive analysis, radiographic progression of structural joint damage at week 24 was reduced with baricitinib versus placebo.

The efficacy of upadacitinib in csDMARDs-IR was evaluated in three RCTs: the SELECT compare, the SELECT next and the SELECT monotherapy.^22^ In the SELECT monotherapy, patients were randomly assigned 2:2:1:1 to switch to once-daily monotherapy of upadacitinib or to continue methotrexate at their existing dose as blinded study drug; starting from week 14, patients assigned to continue methotrexate were switched to 15 mg or 30 mg once-daily upadacitinib per prespecified random assignment at baseline. The primary endpoints in this report are proportion of patients achieving ACR20 at week 14, and proportion achieving low disease activity defined as DAS28[CRP] of 3.2 or lower, both with non-responder imputation at week 14. At week 14, an ACR20 response was achieved by 89 (41%) of patients in the continued methotrexate group, 68% of patients receiving upadacitinib 15 mg, and 71% of patients receiving upadacitinib 30 mg (p<0.0001 for both doses vs continued methotrexate). DAS28(CRP) 3.2 or lower was met by 19% in the continued methotrexate group, 45% of those receiving upadacitinib 15 mg, and 53% of those receiving upadacitinib 30 mg (p<0.0001 for both doses vs continued methotrexate).

In the SELECT next, patients had to have tried at least one csDMARDs for at least 3 months. Patients were randomized to 2 different doses of upadacitinib (15 and 30mg) or placebo, with background csDMARDs.^23^ The primary endpoints were the proportion of patients at week 12 who achieved ACR20, and a DAS28[CRP] of 3.2 or less. 661 were recruited and randomly assigned to receive upadacitinib 15 mg (n=221), upadacitinib 30 mg (n=219), or placebo (n=221). At week 12, ACR20 was achieved by 64% of patients receiving upadacitinib 15 mg and 66% of patients receiving upadacitinib 30 mg, compared with 36% of patients receiving placebo (p<0.0001 for each dose vs placebo). DAS28(CRP) of 3.2 or less was met by 48% of patients receiving upadacitinib 15 mg and 48% of patients receiving upadacitinib 30 mg, compared with 17% of those receiving placebo (p<0.0001 for each dose vs placebo).

In the SELECT compare, similarly to the respective trials with tofacitinib and baricitinib, upadacitinib was compared to adalimumab.^24^ In total, 1.629 RA patients with an inadequate response to MTX were randomized (2:2:1) to receive upadacitinib (15 mg once daily), placebo, or adalimumab (40 mg every other week) while continuing to take a stable background dose of MTX. The primary end points were achievement of an ACR20 improvement response and a DAS28-CRP of <2.6 in the upadacitinib group compared to the placebo group at week 12. Inhibition of radiographic progression was evaluated at week 26. The study was also designed and powered to test for the noninferiority and superiority of upadacitinib compared to adalimumab, as measured both clinically and functionally. At week 12, ACR20 was achieved by 71% of patients in the upadacitinib group compared to 36% in the placebo group and compared to 63% receiving adalimumab (P ≤ 0.05). A DAS28- CRP score of <2.6 at week 12 was achieved by 29% of patients receiving upadacitinib, which was superior to that in the placebo group, in which 6% had a DAS28- CRP score of <2.6 (P ≤ 0.001), and to that in the adalimumab group, in which 18% had a DAS28- CRP score of <2.6 (P ≤ 0.001). Upadacitinib was also superior to adalimumab based on the ACR50 response rate, achievement of a DAS28-CRP score of ≤3.2, change in pain severity score, and change in HAQ. At week 26, more patients receiving upadacitinib than those receiving placebo or adalimumab achieved low disease activity or remission (P ≤ 0.001). Radiographic progression was significantly inhibited in patients receiving upadacitinib and was observed in fewer upadacitinib-treated patients than placebo-treated patients (P ≤ 0.001).

**Figure 3.**
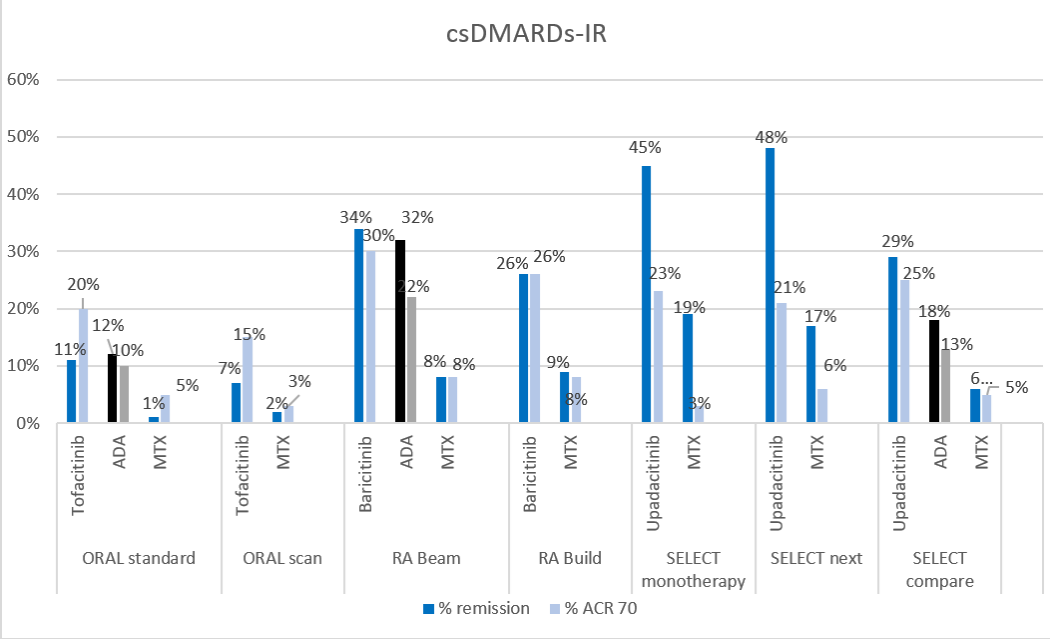
ACR70 response and remission rates (based on DAS28<2.6) for tofacitinib, baricitinib and upadacitinib in csDMARDs-IR (inadequate responders) RA patients. The JAK inhibitors were associated with significant clinical efficacy compared to methotrexate (MTX) and in some of these trials compared also to a bDMARD (ADA=adalimumab).

In *Figure 3*, the rates of remission and ACR70 for the three JAK inhibitors in csDMARD-IR RA patients are summarized.

### 3. After one or more bDMARDs failure

As we mentioned at the beginning, a substantial percentage of patients will fail the first bDMARD for various reasons. Some patients will fail subsequent bDMARDs. It is therefore crucial to assess the efficacy of new drugs, such as the JAK inhibitors, in this difficult to treat RA group.

Two RCTs evaluated the efficacy and safety of tofacitinib in a RA population that have failed csDMARDs and a smaller proportion of patients had failed bDMARDs as well. ORAL Sync was a 12-month trial designed to evaluate the efficacy and safety of tofacitinib in patient with inadequate response to at least one DMARD.^25^ Patients were required to have an inadequate response to treatment with 1 or more stably dosed nonbiologic or biologic DMARDs before baseline and to continue treatment with 1 or more background nonbiologic DMARDs at stable doses throughout the study. Around 6% of patients had already tried an anti-TNF. A total of 792 patients were randomized to two different doses of tofacitinib or placebo in combination with various background cs DMARDs, the majority of whom were on MTX. Primary efficacy outcome measures were an improvement in ACR20 responses at month 6; the change from baseline to month 3 in HAQ-DI; and the percentage of patients meeting DAS28-4(ESR) remission criteria (<2.6) at month 6. Tofacitinib yielded statistically significant improvement in ACR20 response rates with a mean treatment difference for the 5 mg bd group compared with the combined placebo groups of 21.2% (95% CI, 12.2, 30.3%; *P* < 0.001). Tofacitinib demonstrated a rapid onset of benefit with significant ACR20 and ACR50 response rates observed by week 2 and ACR70 by week 4. The HAQ-DI scores at month 3 and DAS28-4(ESR) <2.6 response rates at month 6 were also superior in the tofacitinib groups *vs* placebo. Over time, statistically significant response rates were observed for ACR20 and ACR50 by week 2 in both tofacitinib groups and for ACR70 by week 2 and month 1. Mean treatment differences in changes from baseline in HAQ-DI, DAS28-4(ESR), DAS28-3(C-reactive protein), DAS28-4(ESR) less than 2.6, and Functional Assessment of Chronic Illness Therapy-Fatigue response rates for both tofacitinib groups compared with placebo were also statistically significant over time.

ORAL Solo was a 6-month RCT designed to evaluate the efficacy and safety of tofacitinib monotherapy in adults with active RA who had had an inadequate response (IR) to at least one csDMARD or bDMARD (DMARD-IR) and had discontinued all DMARDs except stable doses of antimalarial agents.^26^ 611 patients were randomized to tofacitinib or placebo. Tofacitinib treatment was associated with statistically significant improvement in the co-primary end points of ACR20 (26.7% in placebo *vs* 59.8% for tofacitinib 5 mg bd; *P* < 0.001) and HAQ-DI (−0.19 in placebo *vs* −0.5 for tofacitinib 5 mg bd; *P* < 0.001) scores at month 3. There were also statistically significant improvements in ACR50 and ACR70 response criteria. The percentage of patients with a DAS28-4(ESR) < 2.6 was not significantly higher with tofacitinib than with placebo (5.6% in the 5-mg group and 4.4% with placebo; P=0.62). Tofacitinib monotherapy in DMARD-IR patients resulted in statistically significant and clinically meaningful improvements in multiple PROs versus placebo at month 3, with sustained improvements over 6 months.^27^

Last but not least, tofacitinib in bDMARD-IR was also assessed in ORAL-step, a 6-month, double-blind, parallel-group phase 3 study.^28^ 399 patients with moderate-to-severe RA and inadequate anti-TNF were randomly assigned to tofacitinib bd 5 mg (n=133); tofacitinib bd 10 mg (n=134); or placebo (n=132), all with background methotrexate. Primary endpoints included ACR20 response rate, mean change from baseline in HAQ-DI, and rates of DAS28<2.6, all at month 3. In this treatment-refractory patient population, in which a third of patients had previously been treated with two or more TNFi, tofacitinib 5 and 10 mg twice a day had rapid, significant, and clinically meaningful improvements compared with placebo. Two out of three co-primary endpoints were met. At month 3, ACR20 response rates were 41.7% for tofacitinib 5 mg versus 24.4% (32 of 131) for placebo (p=0·0024). Improvements from baseline in HAQ-DI were −0·43 for 5 mg tofacitinib versus −0.18 for placebo; DAS28<2.6 rates were 6.7% for 5 mg twice a day tofacitinib versus 1.7% for placebo.

Baricitinib was shown to be associated to significant improvements in clinical outcomes in patients with moderately to severely active RA who had inadequate responses to bDMARDs, including at least one anti-TNF, or had unacceptable side effects, in RA-Beacon.^29^ Significantly more patients receiving baricitinib at the 4-mg dose than those receiving placebo had an ACR20 response at week 12 (55% vs. 27%, P<0.001). Differences between the higher-dose baricitinib group and the placebo group were also significant for the HAQ-DI score and the DAS28-CRP but not for an SDAI score of 3.3 or less.

Upadacitinib was tested in RA patients with active RA and previous inadequate response or intolerance to bDMARDs, with background csDMARDs in the SELECT beyond.^30^ Patients were randomly assigned to receive once-daily oral upadacitinib 15 mg or 30 mg or placebo for 12 weeks, followed by upadacitinib 15 mg or 30 mg from week 12 onwards. The two separate primary endpoints were the proportions of patients achieving a ACR20 response at week 12 and the proportion of patients achieving a DAS28[CRP] of 3.2 or less at week 12. At week 12, ACR20 was achieved by 65% of patients receiving upadacitinib 15 mg compared with 28% of patients receiving placebo (p<0·0001). DAS28(CRP) of 3.2 or less was achieved by 43% of patients receiving upadacitinib 15 mg versus 14% of patients receiving placebo (p<0·0001).

**Figure 4.**
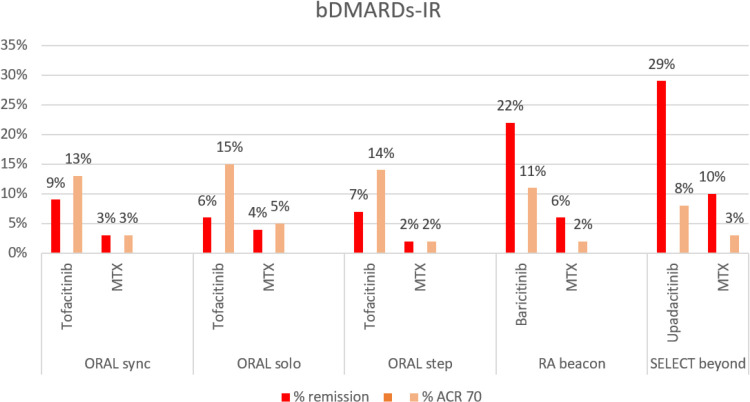
ACR70 response and remission rates (based on DAS28<2.6) for tofacitinib, baricitinib and upadacitinib in RA patients who have failed one or more bDMARDs.

In *Figure 4*, rates of remission and ACR70 for the three JAK inhibitors in refractory, bDMARD-IR RA patients are summarized. Overall worse results are observed, as expected, but still clinically meaningful efficacy in a significant percentage of patients.

## SAFETY OF JAK INHIBITORS

We will not discuss here in detail the safety of JAK inhibitors. The current evidence regarding the safety profile of these drugs is well summarized in a review by Dr. Winthrop.^31^ Additionally, a systematic literature review regarding the safety of bDMARDs and tsDMARDs informing the latest recommendations by EULAR has recently been performed.^32^ Beyond what has already been observed and studied, such as an increased risk of Herpes Zoster infections, a new safety issue, namely thromboembolic events, has emerged for both baricitinib and tofacitinib. For the latter, this risk has been observed for 10mg bd, which is double the dose approved for RA. Patients with a high-risk profile for thromboembolic events are in higher risk during treatment with JAK inhibitors. JAK inhibitors should be used with caution in patients with high risk of such events.^33^

## WHAT IS THE PLACE OF JAK INHIBITORS IN THE TREATMENT ARMAMENTARIUM?

As it is obvious from the summary of phase III RCTs for the three JAK inhibitors approved, they are efficacious both as 1^st^ line therapy, in MTX naïve population, but also after the inadequate response to one or more csDMARDs and, perhaps most importantly, after the failure of one or more bDMARDs. As expected, the efficacy is reduced in the latter patient population, as it is observed with bDMARDs as well. Interestingly, in some studies the JAK inhibitor was shown to be more efficacious than an anti-TNF, like in the RA-beam and SELECT-compare studies. The logical question that follows is where to place this new category of DMARDs in the treatment sequence of RA.

The last update of the EULAR recommendations for the treatment of RA, recommend the addition of a tsDMARD or a bDMARD if the treatment target is not achieved with the first csDMARDs strategy and poor prognostic factors are present.^33^ In contrast to the previous update, there is no preference of bDMARDs over tsDMARDs because of new evidence supporting the successful long-term efficacy and safety of JAK inhibitors.^34–36^ Despite the fact that the 2 abovementioned, non-inferiority trials showed superiority of two JAK inhibitors over an anti-TNF bDMARD, no preference is given to any of these agents for reasons of efficacy.^33^ A recent study using tofacitinib in combination with MTX did not show similar superiority to anti-TNF. The ORAL strategy was a 1 year, double-blind, phase 3b/4, head-to-head, non-inferiority, randomised controlled trial in patients with active RA despite methotrexate therapy. Patients were randomly assigned (1:1:1) to receive tofacitinib monotherapy, tofacitinib + MTX or adalimumab + MTX.^37^ ACR50 response was attained in 38% of patients with tofacitinib monotherapy, 46% of patients with tofacitinib + MTX, and 44% of patients with adalimumab + MTX. Tofacitinib + MTX was non-inferior to adalimumab + MTX, but Non-inferiority could not be claimed for tofacitinib monotherapy versus either adalimumab + MTX or tofacitinib + MTX. Thus, hitherto there is not enough evidence to support preference of tsDMARDs over bDMARDs. This is further supported by recently presented data revealing that another JAK inhibitor that is in phase III trials, filgotinib, does not exhibit superior efficacy when compared with adalimumab.^38^ The choice of DMARD should be guided by current evidence regarding the efficacy, real-life effectiveness, safety but also patient preference (oral over injectable drugs).

Last but not least, it should be highlighted that monotherapy with JAKi can yield excellent clinical results with similar influence to combination therapy on measures of inflammation, and these drugs can be used in cases where use of csDMARDs is contraindicated. However, combination treatment with csDMARDs and JAKi seems to have an advantage in inhibiting radiographic progression compared to JAKi monotherapy, as shown in RA begin trial. Thus, combination should be preferred, when possible.

## CONCLUSIONS

Although dramatic improvements in the field of RA treatment have been achieved, many and important unmet needs continue to exist and pose a challenge for rheumatologists. The JAK inhibitors represent a new class of DMARDs and have proven highly efficacious in different patient populations, even those with refractory disease. Thus, they are an important addition to our therapeutic armamentarium. Their rapid clinical efficacy, the lack of immunogenicity, the oral administration and their short half-life are important advantages. Their safety profile is acceptable and in line with bDMARDs, except for an increased risk herpes zoster infection and a signal for increased risk for thromboembolic events which needs further evaluation. More real-life effectiveness and safety data, as well potential prognostic response factors will improve our understanding for the more optimal use of these drugs in the future.
